# Sublingual NMN administration increases early circulating terminal catabolites 2PY and 4PY compared with oral administration in healthy adult men

**DOI:** 10.1038/s41598-026-57552-9

**Published:** 2026-06-16

**Authors:** Jun Wakabayashi, Seiichiro Higashi, Masashi Morifuji

**Affiliations:** Wellness Science Labs, Meiji Holdings Co., Ltd., 1-29-1 Nanakuni, Hachioji, Tokyo 192-0919 Japan

**Keywords:** Nicotinamide mononucleotide (NMN), Sublingual administration, Bioavailability, Randomized crossover trial, NAD^+^ metabolism, Biochemistry, Drug discovery, Medical research

## Abstract

Sublingual administration of nicotinamide mononucleotide (NMN) is hypothesized to enhance systemic bioavailability by bypassing hepatic first-pass metabolism, a significant limitation of oral intake. However, direct comparative evidence in humans has been lacking. This study aimed to compare the acute pharmacokinetics of NMN following single-dose oral and sublingual administration in a randomized, two-period crossover trial involving 14 healthy adult males. Participants received NMN via both routes, separated by an 8-day washout period. Blood samples were collected for 60 min post-administration to measure NMN and its metabolites, including nicotinamide (NAM), nicotinamide adenine dinucleotide (NAD⁺), and the terminal catabolites N1-methyl-2-pyridone-5-carboxamide (2PY) and N1-methyl-4-pyridone-3-carboxamide (4PY). The incremental area under the curve (iAUC) was significantly higher for the terminal metabolites 2PY and 4PY following sublingual administration compared to the oral route. In contrast, no significant differences were found for the blood concentrations of NMN, NAM, or NAD⁺ between methods. Both administration routes were well-tolerated, with no adverse events reported. These findings provide the first direct evidence in humans that sublingual administration leads to a more rapid increase in the terminal metabolites (2PY and 4PY) over 0–60 min, although the metabolic origin of these increases could not be distinguished in the present study.

**Supplementary Information:**

The online version contains supplementary material available at https://doi.org/10.1038/s41598-026-57552-9.

## Introduction

In today’s era of rapid population aging, a critical goal is to extend “healthspan,” the duration of a healthy life, rather than merely prolonging lifespan. Aging is the single largest risk factor for a wide range of chronic conditions, including cancer, cardiovascular and neurodegenerative diseases, and metabolic disorders^1,2^. In recent years, nicotinamide adenine dinucleotide (NAD^+^), a coenzyme essential for cellular metabolism and signaling, has emerged as a key molecule in efforts to extend healthspan^3–5^.

NAD^+^ is indispensable as a coenzyme for redox reactions in core metabolic pathways and also serves as a critical substrate for enzymes that regulate DNA repair and cellular senescence, including Sirtuins and poly(ADP-ribose) polymerases (PARPs)^6,7^. However, it is well-established that systemic NAD^+^ levels decline with age^8,9^. This decline is believed to contribute significantly to the onset of age-related pathologies by promoting mitochondrial dysfunction and genomic instability^6,10^. A promising approach to restore NAD^+^ levels is the supplementation with NAD^+^ precursors. Nicotinamide mononucleotide (NMN), a direct intermediate in the NAD^+^ biosynthesis pathway, has shown considerable promise^11^. Preclinical studies in animal models have consistently demonstrated that NMN supplementation mitigates various age-associated declines in physiological function, such as weight gain, impaired insulin sensitivity, and reduced physical activity^4,12^. Consequently, NMN is now widely available worldwide as a dietary supplement for anti-aging and health maintenance^13,14^.

Nevertheless, maximizing the benefits of NMN in humans requires a deep understanding of its pharmacokinetics. The majority of NMN supplements are administered orally and are consequently subject to the first-pass effect, wherein the compound is extensively metabolized in the liver after being absorbed from the gastrointestinal tract^15,16^. This raises concerns that the bioavailability of NMN may be compromised, thereby limiting its delivery to peripheral target tissues.

Sublingual administration represents a promising alternative route of administration that bypasses the first-pass effect. The rich vascular network of the sublingual mucosa allows for direct absorption into the systemic circulation, bypassing the gastrointestinal tract and liver. In theory, this route could lead to faster absorption and greater bioavailability than conventional oral intake^17–19^.

Therefore, this study aimed to compare the acute blood kinetics of NMN following oral and sublingual administration in a randomized crossover trial in healthy adult men.

## Results

### Study flow

**Fig. 1:**
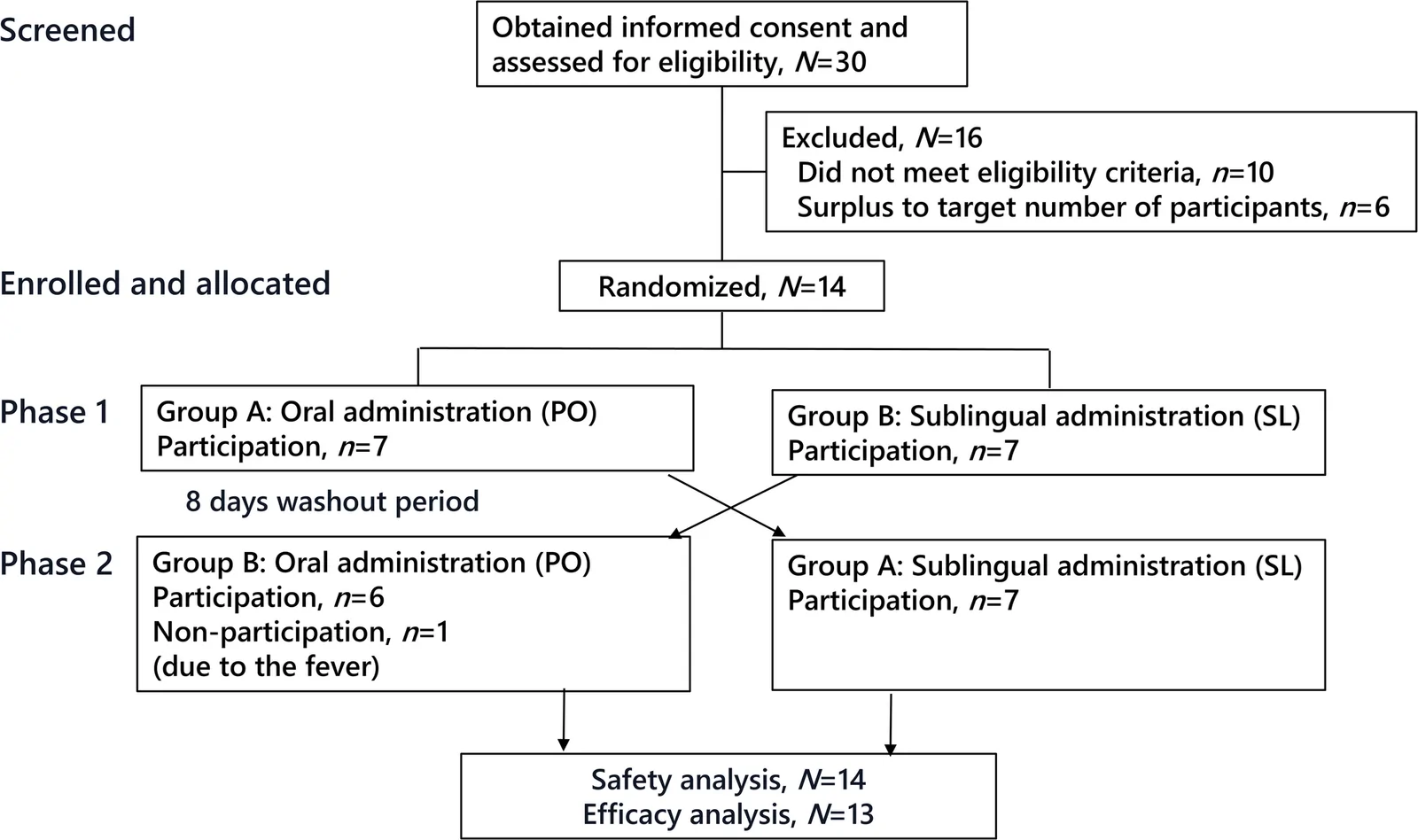
CONSORT flow diagram of the study. A total of 30 individuals were assessed for eligibility. Ten were excluded as they did not meet the eligibility criteria. Of the twenty eligible individuals, fourteen were enrolled because the planned sample size had been reached, and were subsequently enrolled and randomly assigned to one of two treatment sequences: Group A (oral administration followed by sublingual administration) or Group B (sublingual administration followed by oral administration). The two intervention periods were separated by a washout period. One participant assigned to Group B withdrew from the study due to fever after completing the first intervention. Consequently, the safety analysis was conducted on all 14 participants who received at least one intervention, while the efficacy analysis was performed on the 13 participants who completed both periods.

A study flow chart is shown in Fig. 1. A screening test was conducted on 30 participants who consented to participate in the study to determine their eligibility. Ten participants met the exclusion criteria. Of the twenty eligible individuals, fourteen were enrolled because the planned sample size had been reached. One participant in Group B discontinued the study due to fever following the first intervention. Consequently, the safety analysis set comprised all 14 participants, whereas the efficacy analysis set was composed of the 13 participants who completed the trial. Table 1 presents the characteristics of the study participants measured at baseline.

**Table 1:** Presents the characteristics of the study participants measured at baseline.

	Unit			
Number of participants	*N*		13	
Age	year	27	±	4
Height	cm	172.8	±	4.5
Body weight	kg	63.9	±	5.6
Body mass index	kg/m^2^	21.4	±	1.5
Systolic blood pressure	mmHg	118	±	13
Diastolic blood pressure	mmHg	73	±	9
Pulse rate	bpm	73	±	10
White blood cells	/μL	4877	±	1002
Red blood cells	× 10^4^/μL	519	±	36
Hemoglobin	g/L	157	±	7
Hematocrit	L/L	0.480	±	0.024
Platelets	× 10^4^/μL	25.5	±	4.2
Total protein	g/L	75.3	±	2.6
Alkaline phosphatase	IU/L	77.6	±	15.1
Lactate dehydrogenase	IU/L	169	±	20
Aspartate aminotransferase	IU/L	19.7	±	2.7
Alanine aminotransferase	IU/L	17.8	±	6.5
Gamma-glutamyl transpeptidase	IU/L	17.3	±	7.1
Total cholesterol	mmol/L	4.71	±	0.47
Triglycerides	mmol/L	0.986	±	0.501
HDL cholesterol	mmol/L	1.59	±	0.30
LDL cholesterol	mmol/L	2.59	±	0.58
Creatinine	μmol/L	80.4	±	9.9
Uric acid	μmol/L	339	±	56
Glucose	mmol/L	4.85	±	0.24
HbA1c (NGSP)	%	5.2	±	0.2

The values are shown as mean ± standard deviation. (*N* = 13).

### Outcomes

#### Kinetics of blood NMN and related metabolites following oral and sublingual administration of NMN

**Fig. 2:**
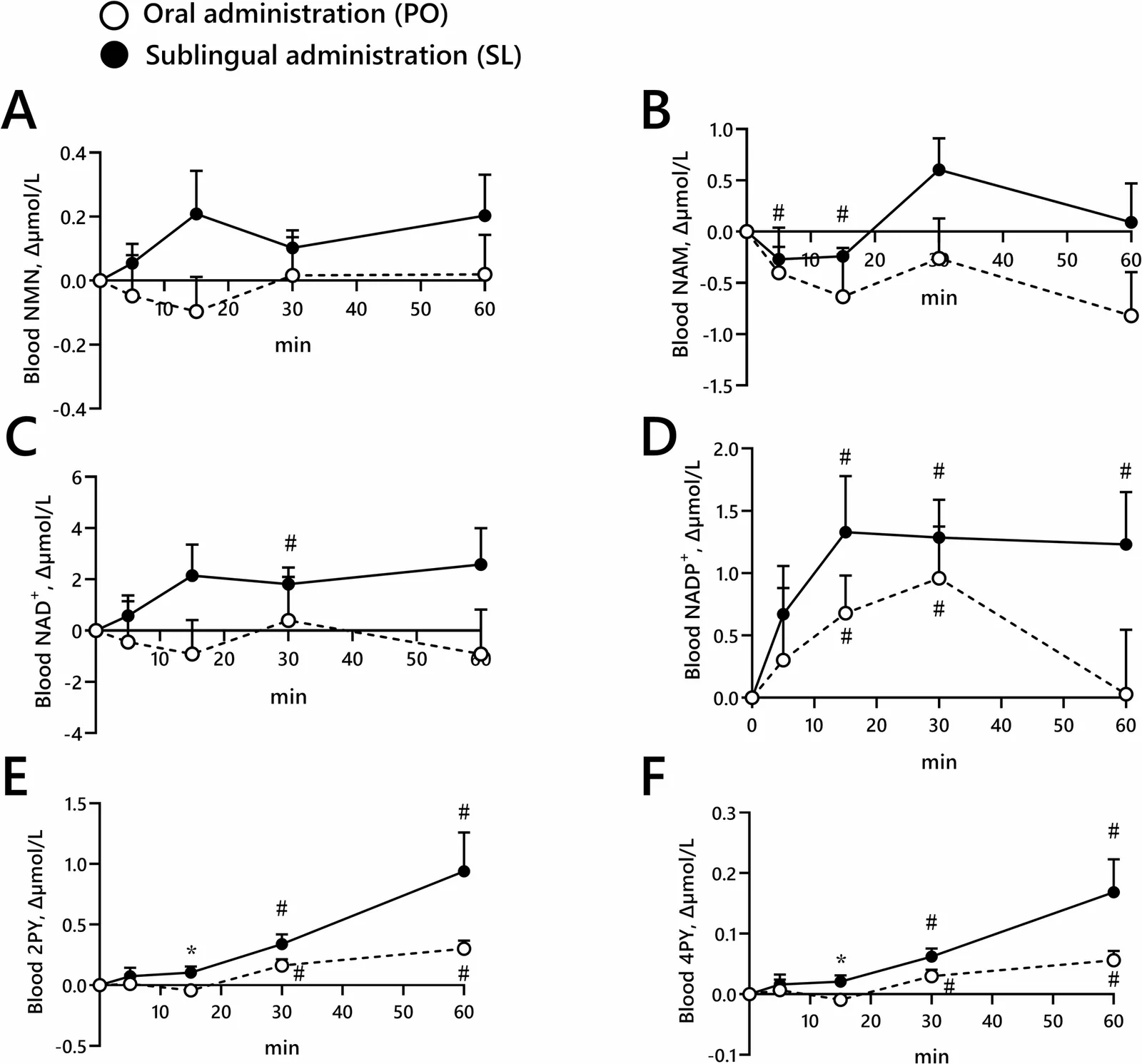
Time-course changes in blood concentrations of NMN and its metabolites. Changes from baseline (Δ) in the blood concentrations of (**A**) NMN, (**B**) NAM, (**C**) NAD^+^, (**D**) NADP^+^, (**E**) 2PY, and (**F**) 4PY are shown following oral (PO) and sublingual (SL) administration of NMN. Blood samples were collected at baseline (0 min) and at 5, 15, 30, and 60 min post-administration. Values are presented as mean ± SEM (*N* = 13). An asterisk (*) indicates a significant difference (*P* < 0.05) between the PO and SL groups at the corresponding time point. A hash symbol (#) indicates a significant difference (*P* < 0.05) from the baseline value (0 min) within each group. Statistical analysis was performed using a repeated-measures two-way ANOVA followed by a post hoc paired t-test. NMN, nicotinamide mononucleotide; NAM, nicotinamide; NAD^+^, nicotinamide adenine dinucleotide; NADP^+^, nicotinamide adenine dinucleotide phosphate; 2PY, N1-methyl-2-pyridone-5-carboxamide; 4PY, N1-methyl-4-pyridone-3-carboxamide.

Figure 2 illustrates the time-course changes in the blood concentrations of NMN, its primary metabolites nicotinamide (NAM), NAD⁺, and nicotinamide adenine dinucleotide phosphate (NADP⁺), and its terminal metabolites N1-methyl-2-pyridone-5-carboxamide (2PY) and N1-methyl-4-pyridone-3-carboxamide (4PY), following NMN administration.

No significant differences were observed between the oral and sublingual administration conditions in the blood concentrations of NMN, NAM, NAD^+^, and NADP^+^ throughout the measurement period. Blood NMN concentrations showed no change from pre-dose levels with either administration method. In the sublingual group, blood NAM concentrations showed a significant decrease at 5 and 15 min post-administration compared to the pre-dose value. In the sublingual group, blood NAD⁺ concentrations were significantly elevated at 30 min post-administration compared to the pre-dose value. Significant increases in blood NADP⁺ concentrations from baseline were observed at 15, 30, and 60 min in the sublingual group, and at 15 and 30 min in the oral group.

Regarding the terminal catabolites of NMN, blood concentrations of 2PY were significantly higher in the sublingual group than in the oral group at 15 min post-administration. Furthermore, compared to baseline values, the sublingual group showed significant increases at 30 and 60 min, whereas the oral group showed a significant increase only at the 30-min time point. Similarly, blood concentrations of 4PY, another terminal catabolite, were also significantly higher in the sublingual group than in the oral group at 15 min post-administration. When compared to baseline, 4PY levels were significantly elevated at 30 and 60 min in both groups.

**Fig. 3:**
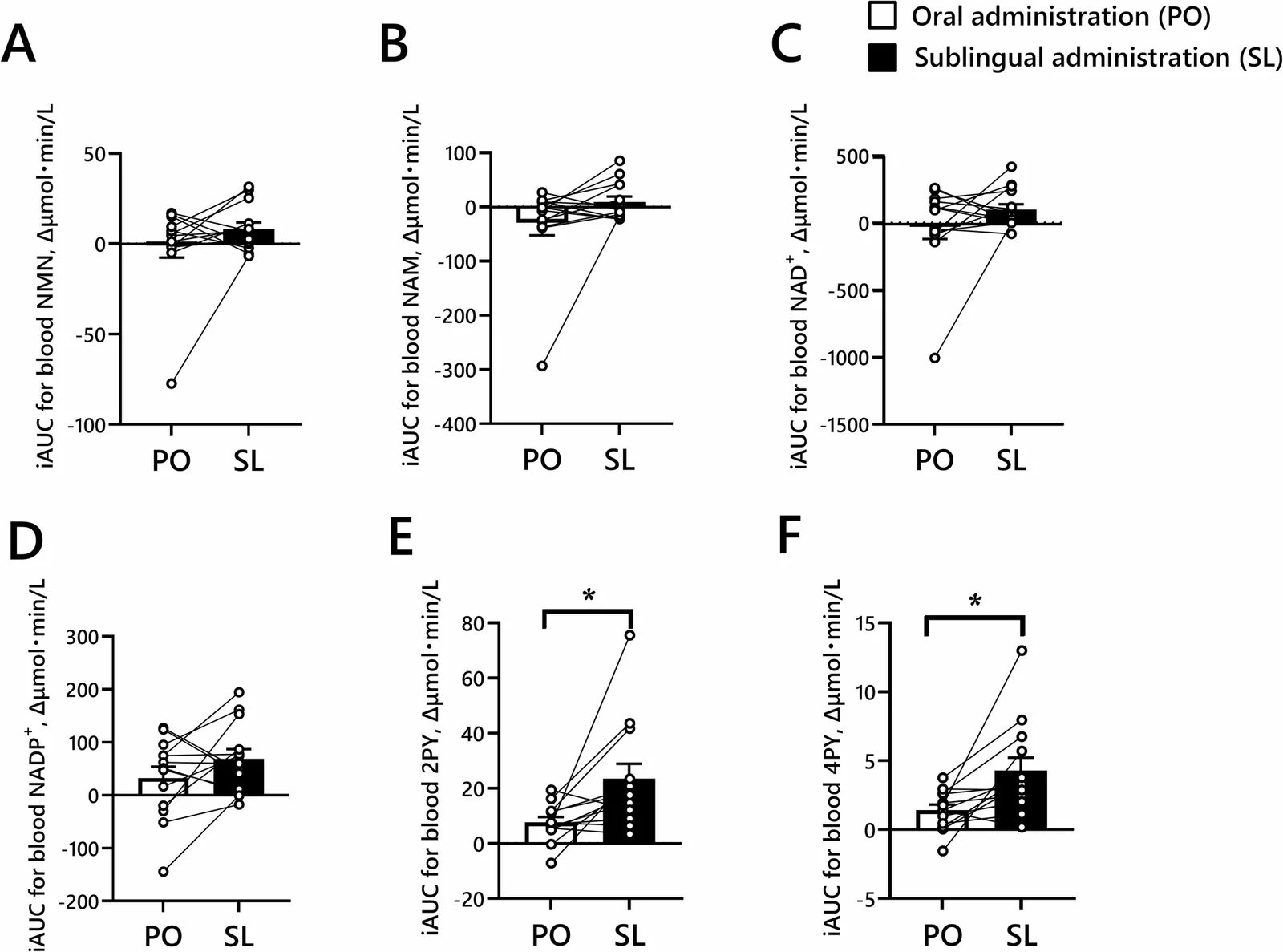
Incremental area under the curve (iAUC) for NMN and its metabolites. The incremental area under the curve (iAUC) from 0 to 60 min was calculated for blood concentrations of (**A**) NMN, (**B**) NAM, (**C**) NAD^+^, (**D**) NADP^+^, (**E**) 2PY, and (**F**) 4PY following oral (PO) and sublingual (SL) administration. The iAUC was calculated using the trapezoidal rule. In each panel, bars represent the mean ± SEM (*N* = 13), and individual data points are shown as open circles. An asterisk (*) indicates a significant difference (*P* < 0.05) between the administration methods, as determined by a paired Student’s t-test. NMN, nicotinamide mononucleotide; NAM, nicotinamide; NAD^+^, nicotinamide adenine dinucleotide; NADP^+^, nicotinamide adenine dinucleotide phosphate; 2PY, N1-methyl-2-pyridone-5-carboxamide; 4PY, N1-methyl-4-pyridone-3-carboxamide.

Calculation of the incremental area under the curve (iAUCs) for each metabolite (Fig. 3) revealed that the iAUCs for both 2PY and 4PY were significantly higher in the sublingual group compared to the oral group. The iAUC for blood NMN and the other metabolites did not differ between administration methods.

### Safety evaluation

No adverse events considered causally related to the consumption of the test substance occurred in this study.

## Discussion

This randomized crossover trial in healthy adult men compared the impact of NMN administration routes on its absorption and metabolic kinetics. Our principal findings are that sublingual NMN administration, relative to conventional oral intake, yielded significantly elevated iAUC over 0–60 min for the blood concentrations of the major terminal metabolites 2PY and 4PY. These findings indicate a greater early systemic exposure to the terminal catabolites 2PY and 4PY after sublingual administration.

The greater early increase in circulating 2PY and 4PY after sublingual administration may be partly explained by partial avoidance of hepatic first-pass metabolism. After oral intake, NMN is absorbed from the gastrointestinal tract and enters the portal circulation before reaching the systemic circulation^15,20^. In contrast, sublingual administration allows absorption through the highly vascularized sublingual mucosa, thereby partially bypassing the liver^17–19^. This difference in the initial route of entry may alter the early systemic handling of NMN-derived metabolites.

However, partial avoidance of hepatic first-pass metabolism may not fully explain the present findings. In our sublingual administration protocol, saliva and any residual material were swallowed after dissolution, and therefore gastrointestinal transit and subsequent microbial conversion could also have contributed to the observed metabolite profiles. In addition, pre-systemic processes in the oral cavity or at the mucosal surface may alter the chemical form of NMN or related precursors before they appear in the circulation. For example, hydrolytic activity at the mucosal surface (e.g., CD38) may generate NAM, whereas microbial deamidation (e.g., nicotinamidase/PncA) may redirect precursors toward the deamidated pathway^21,22^. Notably, PncC has been identified as an NMN deamidase that converts NMN to nicotinic acid mononucleotide (NAMN), supporting a direct microbiota-mediated route from NMN into the deamidated pathway^23,24^. Therefore, the higher 2PY/4PY iAUC observed after sublingual administration should be interpreted as reflecting not only route-dependent absorption, but also potential differences in pre-systemic and downstream metabolic processing. Although the present study evaluated acute kinetics, recent human data suggest that sustained intake of NAD^+^ boosters can influence circulating NAD^+^ and microbial metabolism, supporting the potential relevance of host–microbiome interactions in humans^25^.

**Fig. 4:**
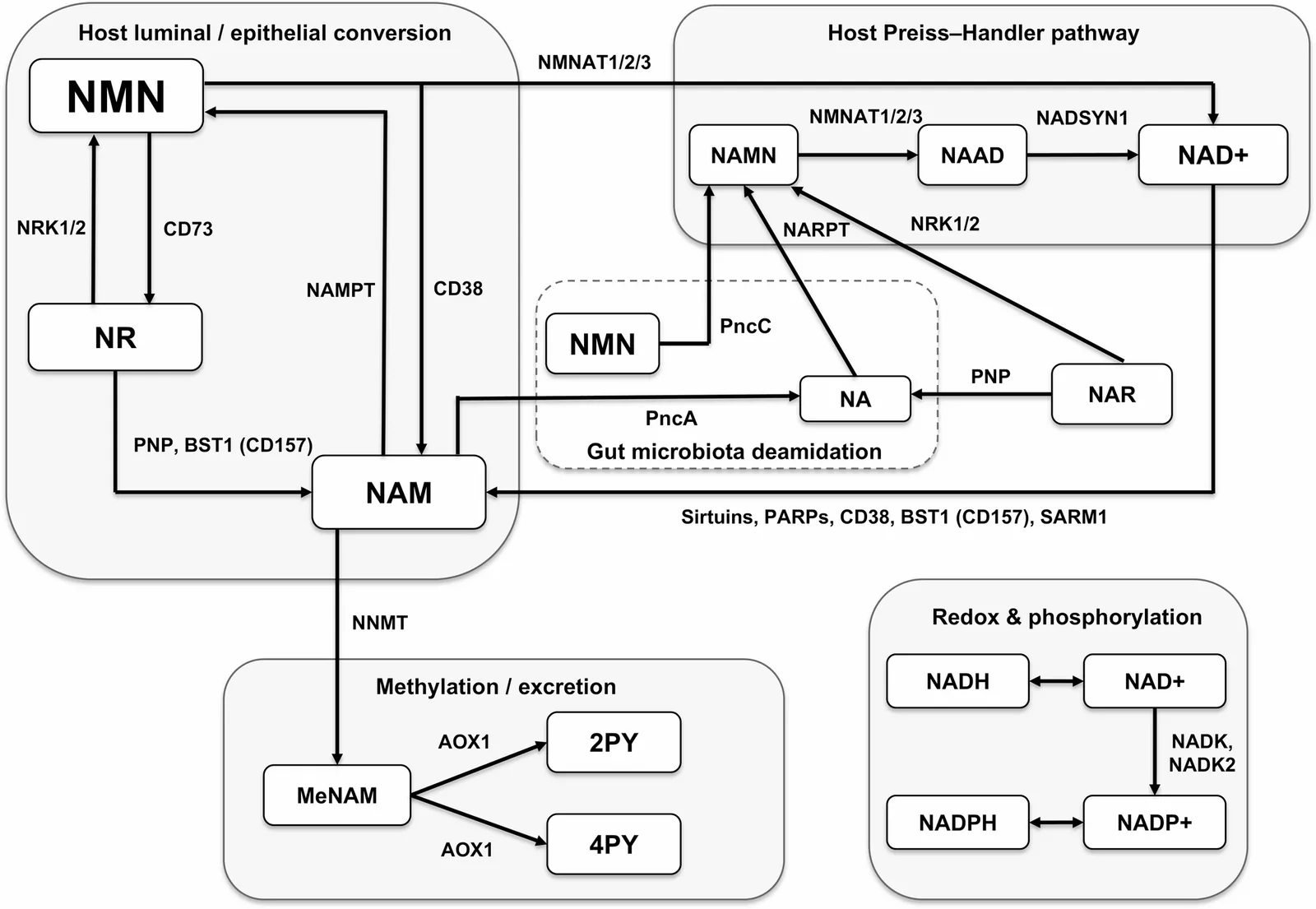
NMN-centered NAD^+^ metabolism integrating host luminal/epithelial conversion, gut microbiota deamidation, and downstream host pathways. Schematic overview of the proposed metabolic fates of nicotinamide mononucleotide (NMN) and its integration with host NAD^+^ biosynthesis and consumption pathways. In the host luminal/epithelial conversion module, extracellular NMN is converted to nicotinamide riboside (NR) by CD73, while NR is phosphorylated back to NMN via NRK1/2. NR is also converted to nicotinamide (NAM) via PNP and/or BST1 (CD157), and NAM is recycled to NMN through NAMPT. In addition, NMN is depicted as being converted to NAM via CD38. In the gut microbiota deamidation module, microbial deamidation contributes not only to nicotinic acid (NA) formation from NAM via nicotinamidase (PncA), but also to the direct conversion of NMN to nicotinic acid mononucleotide (NAMN) via NMN deamidase PncC. Nicotinic acid riboside (NAR) is included as an additional deamidated precursor linked to NA via PNP and to nicotinic acid mononucleotide (NAMN) via NRK1/2. In the host Preiss–Handler pathway, NA is converted to NAMN by NAPRT, then to nicotinic acid adenine dinucleotide (NAAD) by NMNAT1/2/3, and finally to NAD^+^ by NADSYN1. A direct host conversion route from NMN to NAD^+^ via NMNAT1/2/3 is also indicated. NAD^+^ is consumed by sirtuins, PARPs, CD38, BST1 (CD157) and SARM1, regenerating NAM. The figure also summarizes the redox/phosphorylation pathways involving NAD^+^/NADH and NADP^+^/NADPH, and the methylation/excretion pathway in which NAM is converted to MeNAM by NNMT and further oxidized to 2PY and 4PY by AOX1. Boxes indicate metabolites and arrows indicate enzymatic conversions; the dashed enclosure denotes microbiota-mediated conversion. *Abbreviations*: NMN, nicotinamide mononucleotide; NR, nicotinamide riboside; NAM, nicotinamide; NA, nicotinic acid; NAR, nicotinic acid riboside; NAMN, nicotinic acid mononucleotide; NAAD, nicotinic acid adenine dinucleotide; NAD^+^, nicotinamide adenine dinucleotide; NADH, reduced nicotinamide adenine dinucleotide; NADP^+^, nicotinamide adenine dinucleotide phosphate; NADPH, reduced nicotinamide adenine dinucleotide phosphate; MeNAM, 1-methylnicotinamide; AOX1, aldehyde oxidase 1; BST1 (CD157), bone marrow stromal cell antigen 1/cluster of differentiation 157; CD38, NAD^+^ glycohydrolase/cyclic ADP-ribose hydrolase; CD73 (NT5E), ecto-5′-nucleotidase; NADK, NAD kinase; NADK2, mitochondrial NAD kinase; NADSYN1, NAD synthetase 1; NAMPT, nicotinamide phosphoribosyltransferase; NAPRT, nicotinate phosphoribosyltransferase; NMNAT1/2/3, nicotinamide mononucleotide adenylyltransferase 1/2/3; NRK1/2, nicotinamide riboside kinase 1/2; NNMT, nicotinamide N-methyltransferase; PARPs, poly(ADP-ribose) polymerases; PncA, nicotinamidase; PncC, NMN deamidase; PNP, purine nucleoside phosphorylase; SARM1, sterile alpha and TIR motif-containing protein 1; SIRTs, sirtuins.

These possible NMN-centered pathways are summarized schematically in Fig. 4, including additional extracellular host pathways that may influence precursor handling, such as BST1-mediated NR metabolism^26^. In addition to direct intracellular salvage, extracellular conversion of NMN to NR and NAM may reduce the fraction of administered NMN that remains available as intact NMN and may influence downstream NAM catabolism^27–29^. Accordingly, the greater early increase in 2PY and 4PY after sublingual administration may have more than one explanation, including greater delivery of intact precursor to NAD^+^ pools, greater route-dependent conversion to NAM before systemic appearance, or both.

A key limitation of the present study is that the observed increases in circulating terminal catabolites (2PY and 4PY) do not identify their metabolic origin. The present data cannot distinguish among conversion in the oral cavity, gastrointestinal or microbial conversion after swallowing, and hepatic or extrahepatic metabolism. Accordingly, future studies should incorporate direct measurements of intracellular NAD^+^ (for example, in peripheral blood mononuclear cells (PBMCs) or target tissues) and tracer-based pathway analyses to determine whether sublingual administration results in greater intracellular NAD^+^ augmentation than oral administration.

Previous pharmacokinetic studies of NMN in humans have predominantly focused on oral administration. The work of Irie et al. demonstrated that while plasma NMN levels remained largely unchanged after a single oral dose, its metabolites 2PY and 4PY increased time-dependently, corroborating the rapid metabolism of orally ingested NMN and aligning with the findings for our oral cohort^30^. The novelty of the present study, however, resides in being the first to directly compare the pharmacokinetics of sublingual and oral NMN administration in humans, thereby providing quantitative evidence that sublingual administration increases the early systemic appearance of terminal metabolites (2PY/4PY) compared with oral administration.

Nevertheless, certain limitations should be acknowledged. First, our modest sample size (*N* = 14) necessitates caution in generalizing the results, and validation in larger cohorts is warranted. Second, our study population consisted of healthy young adult men; NMN pharmacokinetics may differ between sexes, as well as in elderly individuals or patients with metabolic comorbidities, who constitute key target populations. Third, the observation period was limited to 60 min, and thus we were unable to evaluate NMN metabolism through completion. Moreover, this 0–60-min sampling window precluded the estimation of a full pharmacokinetic profile, including key parameters such as *T*max and total AUC, which are essential for characterizing the complete absorption profile. Fourth, in our sublingual administration protocol, the tablet was allowed to dissolve in saliva and the resulting saliva was subsequently swallowed; therefore, we could not completely exclude the contribution of oral ingestion. In future studies, more stringent approaches will be required to evaluate true sublingual absorption, such as expectoration of the dissolved saliva and/or oral rinsing after dissolution.

These limitations inform several avenues for future research. Larger-scale trials across diverse age groups and health statuses are imperative to establish the robustness of our findings. Critically, future research must move beyond pharmacokinetics to assess whether long-term sublingual NMN supplementation can elicit meaningful improvements in clinical outcomes—such as insulin sensitivity, physical performance, or cognitive function—in the context of placebo-controlled randomized trials.

In conclusion, this study provides the first comparative evidence from a human crossover trial that sublingual NMN administration leads to a faster increase in the major catabolites (2PY and 4PY) than oral ingestion within the 0–60 min. Our findings provide preliminary human evidence relevant to formulation and study design for future NMN research.

## Methods

### Ethical considerations

This study was approved by the Ueno-Asagao Clinic Ethics Review Board on 02/11/2022 (approval number: 2022-38). The board is composed of independent third-party members not involved in the trial. All procedures were conducted in accordance with the ethical principles for medical research involving human subjects as stipulated in the Declaration of Helsinki. The study was registered in the University Hospital Medical Information Network (UMIN) clinical registry system prior to participant enrollment (trial ID: UMIN000049530, registration date: 17/11/2022). The trial was conducted by TES Holdings at the Ueno Asagao Clinic in Tokyo, Japan, and the study period, from screening to the final visit, took place between 11/2022 and 12/2022.

### Study protocol and screening of participants

The investigating clinicians provided prospective participants with a thorough explanation of the study details using the informed consent document. After ensuring that each participant fully understood the information, written informed consent was obtained. Consenting individuals then underwent a screening process. This process included physical and anthropometric measurements (height, body weight, body fat percentage, and body mass index (BMI)), assessment of vital signs (blood pressure and pulse rate), a medical interview conducted by a physician, and the collection of blood and urine samples for analysis. Participants were enrolled in the study if they met all the inclusion criteria and none of the exclusion criteria detailed in Table 2. There was no patient and public involvement (PPI) in the planning, conduct, or reporting of this research.

**Table 2:** Inclusion and exclusion criteria.

Inclusion criteria	
1. Men aged 20 to 35 years at the time of providing informed consent.	
2. Individuals who were fully informed of the study’s purpose and procedures, capable of providing consent, and voluntarily provided written informed consent to participate.	

Exclusion criteria	
1. Use of any medication that could potentially influence the study results within one month prior to the screening, or inability to comply with medication restrictions throughout the study period (excluding occasional use of as-needed medications for conditions like headaches or the common cold).	
2. History of or current psychiatric disorders, sleep disorders, hypertension, diabetes, dyslipidemia, or other serious medical conditions.	
3. History of or current severe diseases of the liver, kidneys, heart, lungs, or hematological system.	
4. History of or current severe gastrointestinal diseases or comorbidities.	
5. Presence of severe anemia.	
6. Body Mass Index (BMI) below 18.5 kg/m^2^ or 25.0 kg/m^2^ and above.	
7. Donation of 200 mL or more of blood within the past month, or 400 mL or more within the past three months.	
8. Habitual consumption of Foods with Function Claims (FFC), health foods, or dietary supplements within the past month, or intention to consume them during the study period.	
9. History of feeling unwell or experiencing adverse reactions during blood collection.	
10. Known allergies to drugs or food components.	
11. Current smokers or individuals who have quit smoking for less than six months.	
12. Daily alcohol intake exceeding 30 g of pure alcohol equivalent.	
13. Current participation in another clinical study or having participated in one within the past month.	
14. Individuals deemed unsuitable for the study by the principal investigator for any other reason.	

### Sample size determination

As this was an exploratory study, the sample size was determined as follows. The sample size was established based on a statistical power analysis with the following parameters: a significance level (α) of 0.05 for a two-sided test, a statistical power (1-β) of 0.80, and an effect size (Cohen’s d) of 0.8, which represents a large effect. The choice of effect size was informed by the lack of previous studies directly comparing sublingual and oral NMN administration. However, sublingual absorption is theoretically expected to result in substantially higher systemic delivery by avoiding the hepatic first-pass effect, a significant barrier for oral administration. Based on this rationale, we anticipated a large difference between the two routes and thus adopted a Cohen’s d of 0.8. The calculation, performed using a statistical analysis software package (BellCurve for Excel, Social Survey Research Information Co., Ltd. Tokyo, Japan), indicated that a minimum of 12 participants would be necessary to achieve the desired power. To accommodate for potential attrition or data loss during the study, the target sample size was set at 14 participants .

### Randomization and allocation

An allocation manager, independent of all other study personnel, used block randomization to assign the 14 eligible participants to one of two treatment sequences (n = 7 per sequence). Participants in Group A received oral administration in the first period, followed by sublingual administration in the second period. Conversely, participants in Group B began with sublingual administration, followed by oral administration.

### Study design

This study was conducted as a randomized, two-period, two-group crossover trial with an 8-day washout period between interventions to minimize carryover effects. On the day before each test, participants consumed a standardized dinner (806 kcal; 131 g of carbohydrate, 29 g of protein, and 18 g of fat) and were instructed to finish the meal by 21:00. Thereafter, they were required to fast, with only the provided water permitted until bedtime; alcohol consumption was prohibited. On the morning of each test day, participants arrived at the facility in a fasted state, with water intake (up to one cup, approx. 200 mL) permitted until two hours prior to arrival. Upon arrival, a compliance check and a physical examination—including measurements of body weight, body fat percentage, BMI, blood pressure, and pulse rate, as well as a medical interview by a physician—were performed prior to the administration of the test substance. Participants were administered one tablet via two different methods. For oral administration, the tablet was swallowed with 100 mL of water after the mouth was rinsed. For sublingual administration, after rinsing the mouth, the tablet was placed under the tongue and held for 3 min to dissolve in saliva, without swallowing. After the 3-min period, the accumulated saliva and any tablet residue were swallowed without water. Blood samples were collected from the median cubital vein at baseline (pre-administration) and at 5, 15, 30, and 60 min post-administration. For both oral and sublingual administration, participants consumed an additional 100 mL of water immediately after the 5-min blood draw.

### Restrictions and prohibitions during the study period

Throughout the study period, spanning from the initial screening to the conclusion of the second observation period, participants were instructed to adhere to a set of guidelines to ensure the integrity of the data. They were required to maintain their usual lifestyle concerning diet, exercise, and sleep, and to avoid irregularities such as sleep deprivation or overeating. Strenuous exercise and lack of sleep were specifically prohibited on the day preceding each testing session. Participants were also directed to refrain from excessive alcohol consumption and to avoid abrupt changes in their drinking habits. The consumption of any health foods, including Foods for Specified Health Uses (FOSHU) and Foods with Function Claims (FFC), as well as smoking, was strictly prohibited. In the event of unavoidable use of medications (including topical agents), quasi-drugs, or Kampo medicines, participants were required to notify the study coordinator and record the product name, manufacturer, and reason for use in a daily diary. This diary was to be completed daily and submitted on scheduled dates. Furthermore, participants were forbidden from enrolling in any other clinical studies or engaging in any other activities that could potentially interfere with the study outcomes.

### Preparation of the test substance

Tablets containing 250 mg of β-NMN per tablet, together with 245 mg of excipient (SWELWiCK®, manufactured by Daicel Corporation) and 5 mg of lubricant (calcium stearate), were prepared. Crystalline NMN (lot no. HNH04-JP210401; purity, 99.9%) was purchased from Japan Bulk Co., Ltd (Osaka, Japan). The purity of NMN was determined using HPLC.

### Evaluations—outcomes

We measured concentrations of NMN (primary outcome), and its metabolites (secondary outcome), NAD^+^, nicotinamide adenine dinucleotide phosphate (NADP^+^), nicotinamide (NAM), N^1^-methyl-2-pyridone-5-carboxamide (2PY), and N^1^-methyl-4-pyridone-3-carboxamide (4PY). As an indicator of bioavailability, the pre-ingestion or baseline (0 min) concentrations were subtracted from subsequent measures to calculate the iAUC.

### Blood and urine analysis

Fasting first-morning urine and blood samples were collected from the median cubital vein. Hematological analysis was performed to measure the following parameters: white blood cell count, red blood cell count, hemoglobin, hematocrit, mean corpuscular volume, mean corpuscular hemoglobin, mean corpuscular hemoglobin concentration, and platelet count. Blood biochemical tests included the assessment of: total protein, creatinine, uric acid, aspartate aminotransferase, alanine aminotransferase, gamma-glutamyl transpeptidase, alkaline phosphatase, lactate dehydrogenase, C-reactive protein, total cholesterol, triglycerides, low-density lipoprotein cholesterol, high-density lipoprotein cholesterol, calculated non-HDL-cholesterol, blood glucose, and hemoglobin A1c. Urinalysis was conducted to evaluate urobilinogen, occult blood, bilirubin, ketone bodies, glucose, protein, pH, and specific gravity. All blood and urine analyses were performed by Hoken Kagaku, Inc. (Kanagawa, Japan).

### Analysis of blood NMN and related metabolites

Levels of NMN and related metabolites (NMN, NAM, NAD^+^, NADP^+^, 2PY and 4PY) were assessed using high-performance liquid chromatography-tandem mass spectrometry (HPLC–MS/MS, HPLC; ACQUITY H-class Bio Binary system, Waters Corporation, Milford, MA, USA, MS/MS; TQ-XS, Waters Corporation). Immediately after collecting blood samples, ice-cold 800 μL methanol was added to 200 μL whole blood. The samples were mixed thoroughly and immediately stored at − 80℃ until analysis. For analysis, mixtures were centrifuged for 10 min at 4℃, 15,000 × *g*. A 40 μL aliquot was mixed with 2 μL internal standard solution and evaporated using a miVac concentrator (Genevac Limited, Gothenburg, Sweden) at 30℃. The dried extract was dissolved with 420 μL citric acid (0.1 mg/mL), and the suspension was filtered through a 0.2 μm filter before HPLC–MS/MS analysis. All analyses were performed on a 2.1 × 150 mm column with a 1.8 μm particle size (ACQUITY UPLC Premier HSS T3, Waters Corporation). Mobile phases A and B were 5 mM ammonium formate and acetonitrile, respectively. The initial eluent composition was 100% A with an increase to 10% B over 3 min. The 10% B concentration was maintained for 0.5 min and then increased to 90% B over 1 min, held at 90% B for 3.5 min, and then reduced to 0% B over 3 min. The total run time was 11 min. The eluent flow was 0.3 mL/min, and the column was maintained at 45℃. Analytes were detected using electrospray ionization in the positive mode. Multiple-reaction-monitoring (MRM) was performed using characteristic fragmentation ions (NAD^+^, m/z = 664.2/428.1, ^13^C^5^ NAD^+^ _IS, m/z = 669.1/428.0; NMN, m/z = 335.2/123.0, NADP^+^, m/z = 744.0/136.0, D^4^ NMN_IS, m/z = 339.1/127.0, NAM, m/z = 123.2/79.9; 2PY, m/z = 153.1/110.0, 4PY, m/z = 153.1/136.0, ^13^C^6^ NAM_IS, m/z = 129.0/85.4). The lower limit of quantification (LLOQ) in human whole blood for this analytical method was 7.5 nM for NMN, 3.8 nM for NAM, 18.8 nM for NAD⁺, 3.8 nM for NADP⁺, 3.8 nM for 2PY, and 3.8 nM for 4PY.

### Safety analysis

Symptom severity and outcome, as well as the frequency of adverse events and side effects that occurred from the start to the end of the study phase for all study participants who had consumed the test substance at least once were recorded. Any associations of the test substance with adverse events were evaluated.

### Statistical analysis

The efficacy analysis population comprised participants who completed all study assessments. The changes in blood NMN-related metabolites over time were analyzed using a two-factor repeated-measures analysis of variance followed by post hoc paired Student’s t-test. The iAUC over 0–60 min was calculated using the trapezoidal rule. Blood NMN and its metabolites were baseline-corrected using fasting values. The iAUC data for NMN and its metabolites in blood were analyzed using the paired Student’s t-test. Differences between treatments were significant at *P* < 0.05. Statistical analyses were performed using SPSS version 26 (IBM Corp., Armonk, NY, USA).

## Supplementary Information

Below is the link to the electronic supplementary material.


Supplementary Material 1



Supplementary Material 2


## Data Availability

The datasets used and/or analyzed during the current study are available from the corresponding author on reasonable request.

## References

[1] Lopez-Otin, C., Blasco, M. A., Partridge, L., Serrano, M. & Kroemer, G. The hallmarks of aging. *Cell* **153**, 1194–1217. 10.1016/j.cell.2013.05.039 (2013). 23746838 PMC3836174

[2] McHugh, D. & Gil, J. Senescence and aging: Causes, consequences, and therapeutic avenues. *J. Cell. Biol.* **217**, 65–77. 10.1083/jcb.201708092 (2018). 29114066 PMC5748990

[3] Igarashi, M. et al. Chronic nicotinamide mononucleotide supplementation elevates blood nicotinamide adenine dinucleotide levels and alters muscle function in healthy older men. *NPJ Aging* **8**, 5. 10.1038/s41514-022-00084-z (2022). 35927255 PMC9158788

[4] Mills, K. F. et al. Long-term administration of nicotinamide mononucleotide mitigates age-associated physiological decline in mice. *Cell Metab.* **24**, 795–806. 10.1016/j.cmet.2016.09.013 (2016). 28068222 PMC5668137

[5] Mouchiroud, L. et al. The NAD(+)/sirtuin pathway modulates longevity through activation of mitochondrial UPR and FOXO signaling. *Cell* **154**, 430–441. 10.1016/j.cell.2013.06.016 (2013). 23870130 PMC3753670

[6] Covarrubias, A. J., Perrone, R., Grozio, A. & Verdin, E. NAD(+) metabolism and its roles in cellular processes during ageing. *Nat. Rev. Mol. Cell. Biol.* **22**, 119–141. 10.1038/s41580-020-00313-x (2021). 33353981 PMC7963035

[7] Migaud, M. E., Ziegler, M. & Baur, J. A. Regulation of and challenges in targeting NAD(+) metabolism. *Nat. Rev. Mol. Cell. Biol.* **25**, 822–840. 10.1038/s41580-024-00752-w (2024). 39026037 PMC12456757

[8] Camacho-Pereira, J. et al. CD38 dictates age-related NAD decline and mitochondrial dysfunction through an SIRT3-dependent mechanism. *Cell Metab.* **23**, 1127–1139. 10.1016/j.cmet.2016.05.006 (2016). 27304511 PMC4911708

[9] Yoshida, M. et al. Extracellular vesicle-contained eNAMPT delays aging and extends lifespan in mice. *Cell Metab.* **30**, 329–342. 10.1016/j.cmet.2019.05.015 (2019). 31204283 PMC6687560

[10] Bhasin, S., Seals, D., Migaud, M., Musi, N. & Baur, J. A. Nicotinamide adenine dinucleotide in aging biology: potential applications and many unknowns. *Endocr. Rev.* **44**, 1047–1073. 10.1210/endrev/bnad019 (2023). 37364580 PMC12102727

[11] D’Angelo, I. et al. Structure of nicotinamide mononucleotide adenylyltransferase: A key enzyme in NAD(+) biosynthesis. *Structure* **8**, 993–1004. 10.1016/s0969-2126(00)00190-8 (2000). 10986466

[12] Yoshino, J., Mills, K. F., Yoon, M. J. & Imai, S. Nicotinamide mononucleotide, a key NAD(+) intermediate, treats the pathophysiology of diet- and age-induced diabetes in mice. *Cell Metab.* **14**, 528–536. 10.1016/j.cmet.2011.08.0144 (2011). 21982712 PMC3204926

[13] Morifuji, M., Higashi, S., Ebihara, S. & Nagata, M. Ingestion of beta-nicotinamide mononucleotide increased blood NAD levels, maintained walking speed, and improved sleep quality in older adults in a double-blind randomized, placebo-controlled study. *Geroscience* **46**, 4671–4688. 10.1007/s11357-024-01204-1 (2024). 38789831 PMC11336149

[14] Kim, M. et al. Effect of 12-week intake of nicotinamide mononucleotide on sleep quality, fatigue, and physical performance in older Japanese adults: A randomized, double-blind placebo-controlled study. *Nutrients*10.3390/nu14040755 (2022). 35215405 PMC8877443

[15] Benjamin, C. & Crews, R. Nicotinamide mononucleotide supplementation: Understanding metabolic variability and clinical implications. *Metabolites*10.3390/metabo14060341 (2024). 38921475 PMC11205942

[16] Liu, L. et al. Quantitative analysis of NAD synthesis-breakdown fluxes. *Cell Metab.* **27**, 1067–1080. 10.1016/j.cmet.2018.03.018 (2018). 29685734 PMC5932087

[17] Bahraminejad, S. & Almoazen, H. Sublingual and buccal delivery: A historical and scientific prescriptive. *Pharmaceutics*10.3390/pharmaceutics17081073 (2025). 40871092 PMC12389210

[18] Liu, J. et al. Biomaterial-based drug delivery strategies for oral mucosa. *Colloids Surf. B Biointerfaces* **251**, 114604. 10.1016/j.colsurfb.2025.114604 (2025). 40081256

[19] Bacskay, I. et al. Bioavailability enhancement and formulation technologies of oral mucosal dosage forms: A review. *Pharmaceutics*10.3390/pharmaceutics17020148 (2025). 40006515 PMC11859484

[20] Sauve, A. A. et al. Triple-isotope tracing for pathway discernment of NMN-induced NAD(+) biosynthesis in whole mice. *Int. J. Mol. Sci.* 10.3390/ijms241311114 (2023). 37446292 PMC10342116

[21] Shats, I. et al. Bacteria boost mammalian host NAD metabolism by engaging the deamidated biosynthesis pathway. *Cell Metab.* **31**, 564–579. 10.1016/j.cmet.2020.02.001 (2020). 32130883 PMC7194078

[22] Yaku, K. et al. Nicotinamide riboside and nicotinamide mononucleotide facilitate NAD(+) synthesis via enterohepatic circulation. *Sci Adv* **11**, eadr1538. 10.1126/sciadv.adr1538 (2025). 40117359 PMC11927621

[23] Kim, L. J. et al. Host-microbiome interactions in nicotinamide mononucleotide (NMN) deamidation. *FEBS Lett.* **597**, 2196–2220. 10.1002/1873-3468.14698 (2023). 37463842

[24] Galeazzi, L. et al. Identification of nicotinamide mononucleotide deamidase of the bacterial pyridine nucleotide cycle reveals a novel broadly conserved amidohydrolase family. *J. Biol. Chem.* **286**, 40365–40375. 10.1074/jbc.M111.275818 (2011). 21953451 PMC3220592

[25] Christen, S. et al. The differential impact of three different NAD(+) boosters on circulatory NAD and microbial metabolism in humans. *Nat. Metab.* **8**, 62–73. 10.1038/s42255-025-01421-8 (2026). 41540253 PMC12855009

[26] Yaku, K. et al. BST1 regulates nicotinamide riboside metabolism via its glycohydrolase and base-exchange activities. *Nat. Commun.* **12**, 6767. 10.1038/s41467-021-27080-3 (2021). 34799586 PMC8604996

[27] Ratajczak, J. et al. NRK1 controls nicotinamide mononucleotide and nicotinamide riboside metabolism in mammalian cells. *Nat. Commun.* **7**, 13103. 10.1038/ncomms13103 (2016). 27725675 PMC5476803

[28] Grozio, A. et al. CD73 protein as a source of extracellular precursors for sustained NAD+ biosynthesis in FK866-treated tumor cells. *J. Biol. Chem.* **288**, 25938–25949. 10.1074/jbc.M113.470435 (2013). 23880765 PMC3764798

[29] Kropotov, A. et al. Purine nucleoside phosphorylase controls nicotinamide riboside metabolism in mammalian cells. *J. Biol. Chem.* **298**, 102615. 10.1016/j.jbc.2022.102615 (2022). 36265580 PMC9667316

[30] Irie, J. et al. Effect of oral administration of nicotinamide mononucleotide on clinical parameters and nicotinamide metabolite levels in healthy Japanese men. *Endocr. J.* **67**, 153–160. 10.1507/endocrj.EJ19-0313 (2020). 31685720

