# Supplementary material for: Sublingual NMN administration increases early circulating terminal catabolites 2PY and 4PY compared with oral administration in healthy adult men
Source: Sci Rep. 2026 Jun 16;16:27464. doi: 10.1038/s41598-026-57552-9 (PMC13534420; doi:10.1038/s41598-026-57552-9)
Supplement: Supplementary file 1 [file 41598_2026_57552_MOESM1_ESM.docx]

**Supplemental information**

**Sample size estimation for an appropriate primary outcome (NMN metabolite 2PY)**

To determine an appropriate sample size when the incremental area under the curve (iAUC) of the NMN terminal metabolite N1-methyl-2-pyridone-5-carboxamide (2PY) is set as the primary outcome, we performed a priori power calculation based on a paired (within-subject) comparison in this randomized two-period crossover design. Specifically, the primary comparison was defined as the within-participant difference in 2PY iAUC (0–60 min) between sublingual and oral administration (i.e., *iAUC*_SL_ − *iAUC*_PO_).

Pilot crossover data (n = 13 completers) were summarized as follows: the mean (SD) 2PY iAUC was 9.64 (4.81) for oral administration and 24.02 (19.32) for sublingual administration. The paired difference (*SL − PO*) had a mean of 14.37 with an SD of 19.89. Based on these paired differences, the standardized effect size for a matched-pairs design (Cohen’s *d*_z_) was calculated as the mean of the paired differences divided by the SD of the paired differences: *d*_z_ = 14.37 / 19.89 = 0.723.

Using this estimated effect size (*d*_z_ ≈ 0.723), we calculated the required sample size for a two-sided paired test with a significance level of α = 0.05 and 80% power (1 − β = 0.80). Under these assumptions, the estimated minimum number of paired observations (i.e., participants completing both periods) was 18 (rounded up from 17.05). To account for potential dropouts or missing data, which are common in crossover studies, we additionally inflated this estimate: assuming 10% attrition yields approximately 20 participants (18/0.9), and assuming 15% attrition yields approximately 22 participants (18/0.85). Accordingly, a practical recruitment target would be 20–22 participants to ensure adequate power for the primary endpoint.
