# Supplementary material for: Sublingual NMN administration increases early circulating terminal catabolites 2PY and 4PY compared with oral administration in healthy adult men
Source: Sci Rep. 2026 Jun 16;16:27464. doi: 10.1038/s41598-026-57552-9 (PMC13534420; doi:10.1038/s41598-026-57552-9)
Supplement: Supplementary file 2 [file 41598_2026_57552_MOESM2_ESM.docx]

**Supplemental Table**

**Blood concentrations of (A) NMN, (B) NAM, (C) NAD^+^, (D) NADP^+^, (E) 2PY, and (F) 4PY**

(A) **Nicotinamide mononucleotide (NMN), unit μM**

| **Oral administration** | | | | | | |
| --- | --- | --- | --- | --- | --- | --- |
| ID | Group | Before, 0 min | 5 min | 15 min | 30 min | 60 min |
| 001 | A | 1.549 | 1.646 | 1.591 | 1.908 | 1.931 |
| 002 | A | 1.932 | 2.008 | 1.926 | 2.065 | 2.073 |
| 003 | A | 1.563 | 1.593 | 1.587 | 1.677 | 1.65 |
| 004 | A | 1.473 | 1.606 | 1.431 | 1.616 | 1.699 |
| 005 | A | 1.576 | 1.609 | 1.656 | 1.981 | 2.012 |
| 006 | A | 1.766 | 1.82 | 1.701 | 1.985 | 2.096 |
| 007 | A | 1.755 | 2.431 | 1.823 | 1.962 | 2.123 |
| 008 | B | 2.027 | 2.074 | 2.017 | 1.984 | 1.99 |
| 009 | B | 1.882 | 1.799 | 1.902 | 1.919 | 1.723 |
| 010 | B | 1.899 | 1.646 | 1.853 | 1.876 | 1.764 |
| 012 | B | 1.724 | 1.706 | 1.777 | 1.77 | 1.697 |
| 013 | B | 1.92 | 1.917 | 1.937 | 1.866 | 1.856 |
| 014 | B | 2.926 | 1.518 | 1.541 | 1.591 | 1.632 |
| **Sublingual administration** | | | | | | |
| ID | Group | Before, 0 min | 5 min | 15 min | 30 min | 60 min |
| 001 | A | 1.564 | 1.494 | 1.419 | 1.429 | 1.553 |
| 002 | A | 1.91 | 2.066 | 3.64 | 1.959 | 2.005 |
| 003 | A | 1.536 | 1.726 | 1.618 | 1.707 | 1.585 |
| 004 | A | 1.573 | 1.61 | 1.61 | 1.531 | 1.425 |
| 005 | A | 1.651 | 1.821 | 1.818 | 1.759 | 1.736 |
| 006 | A | 1.331 | 1.871 | 1.86 | 1.949 | 1.669 |
| 007 | A | 1.697 | 1.731 | 1.77 | 1.666 | 1.672 |
| 008 | B | 2.167 | 2.009 | 2.257 | 2.092 | 2.349 |
| 009 | B | 2.006 | 2.131 | 2.022 | 2.245 | 3.668 |
| 010 | B | 1.808 | 1.821 | 1.895 | 2.047 | 2.116 |
| 012 | B | 1.74 | 1.771 | 1.866 | 1.903 | 1.918 |
| 013 | B | 1.898 | 1.965 | 1.91 | 1.966 | 1.925 |
| 014 | B | 1.86 | 1.429 | 1.767 | 1.819 | 1.761 |

(B) **Nicotinamide (NAM), unit μM**

| **Oral administration** | | | | | | |
| --- | --- | --- | --- | --- | --- | --- |
| ID | Group | Before, 0 min | 5 min | 15 min | 30 min | 60 min |
| 001 | A | 1.074 | 0.896 | 1.047 | 0.716 | 1.261 |
| 002 | A | 1.638 | 0.983 | 1.043 | 1.517 | 0.864 |
| 003 | A | 0.975 | 1.834 | 0.86 | 1.222 | 0.874 |
| 004 | A | 1.164 | 1.017 | 1.195 | 1.266 | 1.097 |
| 005 | A | 1.571 | 1.014 | 0.955 | 0.98 | 0.656 |
| 006 | A | 1.064 | 2.079 | 1.355 | 0.95 | 1.239 |
| 007 | A | 1.138 | 1.055 | 1.041 | 1.018 | 0.928 |
| 008 | B | 0.894 | 0.953 | 1.201 | 1.8 | 1.019 |
| 009 | B | 1.845 | 1.461 | 1.212 | 3.529 | 0.703 |
| 010 | B | 1.672 | 2.096 | 0.962 | 1.461 | 0.762 |
| 012 | B | 6.334 | 0.975 | 0.913 | 1.877 | 0.657 |
| 013 | B | 1.582 | 0.969 | 1.028 | 1.047 | 0.704 |
| 014 | B | 1.347 | 1.751 | 1.239 | 1.531 | 0.858 |
| **Sublingual administration** | | | | | | |
| ID | Group | Before, 0 min | 5 min | 15 min | 30 min | 60 min |
| 001 | A | 1.368 | 0.87 | 0.806 | 1.145 | 0.898 |
| 002 | A | 1.404 | 1.136 | 0.993 | 3.538 | 1.399 |
| 003 | A | 1.431 | 0.772 | 1.541 | 2.138 | 0.797 |
| 004 | A | 1.289 | 1.505 | 1.122 | 4.169 | 1.01 |
| 005 | A | 1.65 | 0.673 | 0.857 | 2.25 | 0.945 |
| 006 | A | 1.124 | 1.06 | 1.075 | 2.999 | 1.192 |
| 007 | A | 1.205 | 0.877 | 0.83 | 2.246 | 5.759 |
| 008 | B | 1.09 | 0.724 | 1.276 | 1.012 | 0.793 |
| 009 | B | 1.331 | 2.1 | 0.881 | 0.781 | 0.725 |
| 010 | B | 0.743 | 0.513 | 0.849 | 1.347 | 0.7 |
| 012 | B | 0.902 | 0.521 | 0.684 | 0.602 | 0.656 |
| 013 | B | 0.99 | 0.925 | 0.913 | 0.645 | 0.942 |
| 014 | B | 1.402 | 0.742 | 0.987 | 0.89 | 1.301 |

**(C) Nicotinamide adenine dinucleotide (NAD^+^), unit μM**

| **Oral administration** | | | | | | |
| --- | --- | --- | --- | --- | --- | --- |
| ID | Group | Before, 0 min | 5 min | 15 min | 30 min | 60 min |
| 001 | A | 20.74 | 22.02 | 23.36 | 28.38 | 22.74 |
| 002 | A | 22.24 | 24.78 | 24.76 | 25.87 | 25.43 |
| 003 | A | 20.26 | 21.59 | 22.63 | 23.05 | 23.91 |
| 004 | A | 22.13 | 23.21 | 21.34 | 25.15 | 25.15 |
| 005 | A | 16.83 | 16.91 | 16.25 | 20.57 | 19.51 |
| 006 | A | 21.8 | 21.69 | 20.25 | 25.18 | 24.4 |
| 007 | A | 22.58 | 32.3 | 24.62 | 26.27 | 27.77 |
| 008 | B | 19.99 | 20.47 | 20.14 | 18.69 | 18.6 |
| 009 | B | 23.55 | 22.36 | 24.1 | 23.12 | 19.56 |
| 010 | B | 20.23 | 17.12 | 18.3 | 18.8 | 15.88 |
| 012 | B | 18.31 | 17.21 | 16.64 | 17.23 | 16.03 |
| 013 | B | 21.57 | 21.5 | 21.8 | 20.97 | 18.07 |
| 014 | B | 37.62 | 20.89 | 21.78 | 19.72 | 18.93 |
| **Sublingual administration** | | | | | | |
| ID | Group | Before, 0 min | 5 min | 15 min | 30 min | 60 min |
| 001 | A | 17.7 | 19.72 | 18.12 | 18.33 | 19.42 |
| 002 | A | 21.48 | 23.51 | 36.7 | 22.12 | 22.61 |
| 003 | A | 16.46 | 18.73 | 17.39 | 18.84 | 17.77 |
| 004 | A | 19.7 | 20.4 | 20.89 | 19.2 | 19.19 |
| 005 | A | 14.79 | 16.31 | 14.21 | 15.47 | 15.2 |
| 006 | A | 15.25 | 19.95 | 20.54 | 21.24 | 18.37 |
| 007 | A | 20.05 | 20.14 | 20.93 | 20.41 | 18.92 |
| 008 | B | 25.32 | 22.27 | 22.68 | 24.03 | 25.46 |
| 009 | B | 26.37 | 27.2 | 28.27 | 31.21 | 44.91 |
| 010 | B | 19.51 | 22.31 | 22.45 | 25.32 | 25.06 |
| 012 | B | 19.28 | 19.58 | 21.52 | 21.3 | 22.2 |
| 013 | B | 26.02 | 26.14 | 25.55 | 26.53 | 25.82 |
| 014 | B | 24.77 | 17.95 | 25.4 | 26.3 | 25.34 |

(D) **Nicotinamide adenine dinucleotide phosphate (NADP^+^), unit μM**

| **Oral administration** | | | | | | |
| --- | --- | --- | --- | --- | --- | --- |
| ID | Group | Before, 0 min | 5 min | 15 min | 30 min | 60 min |
| 001 | A | 4.762 | 4.03 | 6.598 | 7.324 | 6.099 |
| 002 | A | 6.2 | 4.459 | 6.26 | 5.436 | 6.151 |
| 003 | A | 5.031 | 3.628 | 5.662 | 5.682 | 5.329 |
| 004 | A | 3.622 | 5.63 | 4.881 | 4.165 | 5.736 |
| 005 | A | 4.328 | 4.303 | 5.872 | 7.483 | 6.768 |
| 006 | A | 3.991 | 6.736 | 4.361 | 5.776 | 4.592 |
| 007 | A | 3.878 | 8.26 | 5.652 | 5.068 | 6.677 |
| 008 | B | 5.021 | 6.338 | 6.189 | 7.025 | 4.498 |
| 009 | B | 5.167 | 4.827 | 5.358 | 5.28 | 3.68 |
| 010 | B | 6.004 | 5.831 | 7.142 | 7.693 | 6.023 |
| 012 | B | 6.265 | 6.522 | 5.617 | 5.413 | 4.533 |
| 013 | B | 7.391 | 8.679 | 8.875 | 9.668 | 5.284 |
| 014 | B | 8.799 | 5.135 | 6.843 | 6.913 | 5.476 |
| **Sublingual administration** | | | | | | |
| ID | Group | Before, 0 min | 5 min | 15 min | 30 min | 60 min |
| 001 | A | 4.206 | 5.385 | 7.173 | 7.034 | 7.672 |
| 002 | A | 4.695 | 6.261 | 9.005 | 7.282 | 6.64 |
| 003 | A | 3.514 | 4.515 | 4.625 | 5.121 | 4.634 |
| 004 | A | 3.228 | 4.748 | 4.918 | 4.471 | 4.348 |
| 005 | A | 4.876 | 5.424 | 7.468 | 5.368 | 4.953 |
| 006 | A | 2.021 | 5.917 | 5.319 | 5.415 | 5.201 |
| 007 | A | 4.802 | 6.067 | 4.953 | 5.534 | 5.678 |
| 008 | B | 5.776 | 4.441 | 6.938 | 6.935 | 7.711 |
| 009 | B | 5.214 | 6.239 | 5.913 | 6.209 | 8.382 |
| 010 | B | 5.167 | 4.842 | 4.836 | 5.566 | 5.489 |
| 012 | B | 6.143 | 6.161 | 4.496 | 7.013 | 5.009 |
| 013 | B | 6.411 | 6.101 | 7.146 | 7.448 | 5.174 |
| 014 | B | 5.441 | 4.102 | 5.97 | 4.814 | 6.57 |

(E) **N1-methyl-2-pyridone-5-carboxamide (2PY), unit μM**

| **Oral administration** | | | | | | |
| --- | --- | --- | --- | --- | --- | --- |
| ID | Group | Before, 0 min | 5 min | 15 min | 30 min | 60 min |
| 001 | A | 0.887 | 0.965 | 0.866 | 0.972 | 1.498 |
| 002 | A | 0.866 | 0.804 | 0.815 | 1.021 | 1.079 |
| 003 | A | 1.408 | 2.286 | 1.196 | 1.279 | 1.322 |
| 004 | A | 1.522 | 1.499 | 1.547 | 1.795 | 1.884 |
| 005 | A | 2.473 | 2.447 | 2.295 | 2.866 | 2.455 |
| 006 | A | 1.27 | 1.299 | 1.299 | 1.466 | 2.018 |
| 007 | A | 0.93 | 0.925 | 0.927 | 0.993 | 1.282 |
| 008 | B | 1.636 | 1.523 | 1.785 | 1.896 | 1.951 |
| 009 | B | 1.303 | 1.059 | 1.056 | 1.16 | 1.368 |
| 010 | B | 1.676 | 1.664 | 1.62 | 1.927 | 1.817 |
| 012 | B | 0.824 | 0.639 | 0.731 | 0.926 | 1.165 |
| 013 | B | 1.391 | 1.321 | 1.477 | 1.894 | 1.889 |
| 014 | B | 1.289 | 1.18 | 1.309 | 1.4 | 1.663 |
| **Sublingual administration** | | | | | | |
| ID | Group | Before, 0 min | 5 min | 15 min | 30 min | 60 min |
| 001 | A | 0.459 | 0.466 | 0.482 | 0.775 | 1.304 |
| 002 | A | 2.518 | 2.369 | 2.233 | 2.686 | 2.802 |
| 003 | A | 0.981 | 0.992 | 1.027 | 1.462 | 1.598 |
| 004 | A | 1.182 | 1.174 | 1.276 | 2.24 | 2.294 |
| 005 | A | 1.342 | 1.304 | 1.423 | 1.654 | 1.803 |
| 006 | A | 2.185 | 2.39 | 2.707 | 2.998 | 3.331 |
| 007 | A | 0.673 | 0.615 | 0.734 | 0.899 | 5.344 |
| 008 | B | 0.997 | 1.025 | 1.262 | 1.238 | 1.61 |
| 009 | B | 1.495 | 2.356 | 1.683 | 1.642 | 2.243 |
| 010 | B | 1.028 | 1.097 | 1.182 | 1.156 | 1.271 |
| 012 | B | 0.872 | 0.884 | 0.953 | 1.188 | 1.495 |
| 013 | B | 1.105 | 1.226 | 1.227 | 1.277 | 1.494 |
| 014 | B | 1.237 | 1.141 | 1.249 | 1.244 | 1.692 |

(F) **N1-methyl-4-pyridone-3-carboxamide (4PY), unit μM**

| **Oral administration** | | | | | | |
| --- | --- | --- | --- | --- | --- | --- |
| ID | Group | Before, 0 min | 5 min | 15 min | 30 min | 60 min |
| 001 | A | 0.208 | 0.233 | 0.212 | 0.226 | 0.338 |
| 002 | A | 0.187 | 0.176 | 0.181 | 0.223 | 0.235 |
| 003 | A | 0.315 | 0.515 | 0.268 | 0.289 | 0.296 |
| 004 | A | 0.345 | 0.345 | 0.356 | 0.404 | 0.425 |
| 005 | A | 0.605 | 0.61 | 0.569 | 0.706 | 0.61 |
| 006 | A | 0.285 | 0.295 | 0.294 | 0.327 | 0.46 |
| 007 | A | 0.211 | 0.215 | 0.221 | 0.229 | 0.291 |
| 008 | B | 0.297 | 0.268 | 0.312 | 0.332 | 0.339 |
| 009 | B | 0.237 | 0.197 | 0.194 | 0.204 | 0.24 |
| 010 | B | 0.3 | 0.3 | 0.288 | 0.342 | 0.313 |
| 012 | B | 0.149 | 0.111 | 0.126 | 0.153 | 0.195 |
| 013 | B | 0.249 | 0.235 | 0.261 | 0.328 | 0.324 |
| 014 | B | 0.245 | 0.215 | 0.237 | 0.25 | 0.289 |
| **Sublingual administration** | | | | | | |
| ID | Group | Before, 0 min | 5 min | 15 min | 30 min | 60 min |
| 001 | A | 0.086 | 0.09 | 0.089 | 0.135 | 0.219 |
| 002 | A | 0.458 | 0.431 | 0.397 | 0.481 | 0.499 |
| 003 | A | 0.173 | 0.175 | 0.181 | 0.249 | 0.271 |
| 004 | A | 0.213 | 0.212 | 0.224 | 0.387 | 0.393 |
| 005 | A | 0.245 | 0.24 | 0.257 | 0.303 | 0.316 |
| 006 | A | 0.389 | 0.431 | 0.488 | 0.542 | 0.585 |
| 007 | A | 0.12 | 0.112 | 0.131 | 0.157 | 0.924 |
| 008 | B | 0.228 | 0.232 | 0.29 | 0.276 | 0.354 |
| 009 | B | 0.331 | 0.53 | 0.376 | 0.38 | 0.5 |
| 010 | B | 0.232 | 0.252 | 0.262 | 0.261 | 0.288 |
| 012 | B | 0.202 | 0.205 | 0.224 | 0.268 | 0.334 |
| 013 | B | 0.246 | 0.277 | 0.275 | 0.285 | 0.339 |
| 014 | B | 0.296 | 0.27 | 0.299 | 0.294 | 0.385 |
